# An Evaluation of the Effects of Photobiomodulation Therapy on the Peri-Implant Bone Healing of Implants with Different Surfaces: An In Vivo Study

**DOI:** 10.3390/ma15134371

**Published:** 2022-06-21

**Authors:** Pier Paolo Poli, Laís Kawamata de Jesus, Ulisses Ribeiro Campos Dayube, Henrique Hadad, Caroline Loureiro, Fernando Yamamoto Chiba, Thayane Silveira Mata Furtado, Maísa Pereira Silva, Roberta Okamoto, Carlo Maiorana, Paulo Sergio Perri de Carvalho, Francisley Ávila Souza

**Affiliations:** 1 Implant Center for Edentulism and Jawbone Atrophies, Maxillofacial Surgery and Odontostomatology Unit, Fondazione IRCCS Cà Granda Ospedale Maggiore Policlinico, 20122 Milan, Italy; carlo.maiorana@unimi.it; 2 Department of Biomedical, Surgical and Dental Sciences, University of Milan, 20122 Milan, Italy; 3 Department of Diagnosis and Surgery, School of Dentistry, São Paulo State University (UNESP), Araçatuba 16 015 050, SP, Brazil; lais.kawamata@unesp.br (L.K.d.J.); henriquehadad@gmail.com (H.H.); maisa.silva@unesp.br (M.P.S.); 4 Implant Dentistry Postgraduate Program, São Leopoldo Mandic School of Dentistry and Research Center, Campinas 13 045 755, SP, Brazil; dayubecd@globo.com (U.R.C.D.); thayane@furtado.com.br (T.S.M.F.); paulo.perri@unesp.br (P.S.P.d.C.); 5 Department of Preventive and Restorative Dentistry, School of Dentistry, São Paulo State University (UNESP), Araçatuba 16 015 050, SP, Brazil; cah__loureiro@hotmail.com (C.L.); fernando.chiba@unesp.br (F.Y.C.); 6 Department of Basic Sciences, School of Dentistry, São Paulo State University (UNESP), Araçatuba 16 015 050, SP, Brazil; roberta.okamoto@unesp.br

**Keywords:** dental implants, osseointegration, photobiomodulation therapy, low-level laser therapy, gallium–aluminum–arsenide, topographic characterization

## Abstract

(1) Background: This study evaluates the effects of photobiomodulation (PBM) therapy on the peri-implant bone healing of implants with a machined surface (MS) and treated surface (TS). (2) Methods: Topographic characterization of the surfaces (scanning electron microscopy [SEM]- energy dispersive X-ray spectroscopy [EDX]) was performed before and after implant removal. Twenty rabbits were randomly divided into four groups: MS and TS groups (without PBM therapy) and LMS and LTS groups (with PBM therapy). After implant placement, the stability coefficient (ISQ) was measured. In the periods of 21 and 42 days, the ISQ was measured again, followed by biomechanical analysis. (3) Results: The surfaces of the TS implants showed topographic differences compared with MS implants. The ISQ values of the LMS were statistically significant when compared with those of the MS at 42 days (*p* < 0.001). The removal torque values of the LMS were statistically significant when compared with those of the MS at 21 days (*p* = 0.023) and 42 days (*p* = 0.023). For SEM, in general, the LMS, TS and LTS presented high bone tissue coverage when compared to MS. (4) Conclusions: The PBM therapy modulated the osseointegration process and was evidenced mainly on the machined surface.

## 1. Introduction

Laser is an English language acronym meaning “light amplification by stimulated emission of radiation” [1]. The action of a laser can differ according to its wavelength and interaction with the target tissue [2,3]. A cell has a survival threshold based on the type of tissue, its locations and its physiological status. When a laser is employed with respect to this threshold, a low energy intensity is offered to the cell, and the laser is operated at a low energy density. Low-level lasers such as helium–neon (He-Ne) [4] and gallium–aluminum–arsenide (GaAlAs) [5] can be activated by different chemical methods.

Given their therapeutic potential, low-level lasers have been used to heal hard and soft tissues. Its action modulates the tissue repair process, promotes anti-inflammatory and analgesic effects, stimulates the immune system, allows cell regeneration and favors the processes of angiogenesis, collagen synthesis and osteogenesis [6,7,8]. The therapeutic effects of low-level lasers for the treatment of wounds were first described in 1971 by Mester et al. [9], followed by several other researchers [10,11,12] who proved the biostimulation effects of laser therapy.

Considering that the therapeutic effects of PBM therapy include accelerating the bone repair process, previously published studies [13,14,15] applied methodologies with different wavelengths and exposure times to modulate and allow the acceleration of the osseointegration process. The application of low-level laser therapy (LLLT) in the surgical site during the osseointegrated implant installation provided a large bone–implant contact, high values of resonance frequency and a removal torque [13,14,15,16,17,18,19,20].

Thus, this study aims to evaluate the process of the peri-implant bone healing of implants with machined surfaces and treated surfaces installed in the tibiae of rabbits treated with or without PBM therapy using frequency analysis by resonance, removal torque and scanning electron microscopy (SEM), coupled to an energy dispersive X-ray spectroscopy (EDX) for implants prior to installation and removed by a removal torque.

## 2. Materials and Methods

### 2.1. Study Design and Ethics

**Figure 1:**
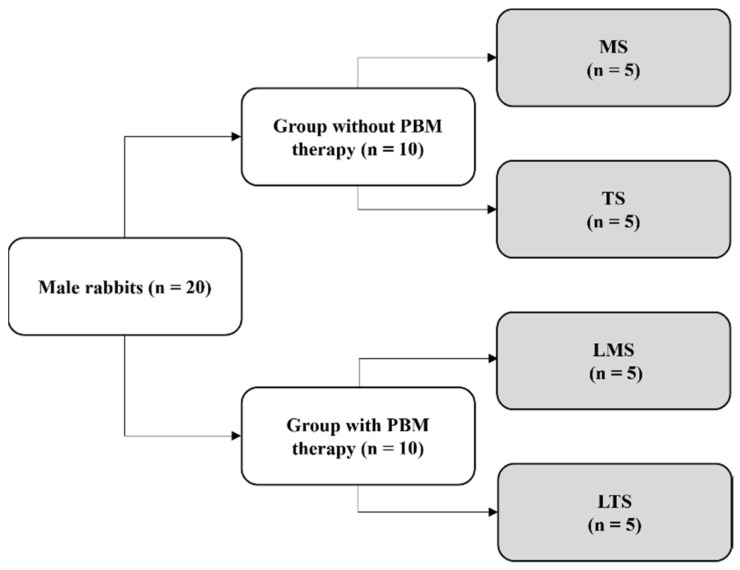
Division of animals into four groups according to the surface of the implant received and the application of PBM therapy.

This experimental study was performed according to the Ethical Principles for Animal Experimentation adopted by the Brazilian Animal Experimentation Board (COBEA) and was approved by the Animal Ethics and Experimentation Committee (CEEA) with the protocol number (protocol n° FOA-00950-2013). The project was designed in compliance with ARRIVE reporting guidelines [21]. Twenty male white rabbits (Albinus) weighing approximately 3 to 4 kg and 5 months of age were kept in individual and acclimatized cages with a standard diet, fed with solid rations (Procoelho, Primor, São Paulo, Brazil) and with ad libitum access to water. These animals randomly received an implant with a machined surface or treated surface (surface sandblasting followed by acid etching) in their right and left tibia (external hexagon connection), manufactured using the Ti-6Al-4V (titanium–6aluminum–4vanadium) alloy (ASTM 67) and measuring 4 mm in diameter and 10 mm in length (Novo Colosso, Emfils Ind Com Produtos Odontológicas, São Paulo, Brazil). An osteotomy was performed in the medial region of the lateral surface of the tibia with standardized drilling for an implant of 3.5/3.8 mm in diameter and 10 mm in depth. The animals were then randomly divided into four groups according to the implant surface received and the application of PBM therapy (Figure 1).

- MS group: Animals with a machined surface implant, which did not receive PBM therapy.
- TS group: Animals with a treated surface implant, which did not receive PBM therapy.
- LMS group: Animals with a machined surface implant, which received PBM therapy.
- LTS group: Animals with a treated surface, which received PBM therapy.

The animals received perpendicular PBM therapy in the osteotomized surgical site before implant placement and immediately after suturing in four different positions.

### 2.2. Implant Placement and PBM Therapy

After fasting for 8 h, general anesthesia was administered by an intramuscular injection of 50 mg/kg ketamine (Vetaset–Fort Dodge Saúde Animal Ltda, São Paulo, Brazil) and 5 mg/kg xylazine (Dopaser–Laboratório Calier do Brazil Ltda, São Paulo, Brazil). For complementary anesthesia, the animals received local anesthesia with mepivacaine (0.3 mL/kg, Scandicaine 2% with adrenaline 1:100,000, Septodont, Saint Maur des Fossé, France) in the region to be operated upon. Then, a 2 cm dermal periosteal incision was made in the tibial metaphysis, and the soft tissue was divulsed, exposing the bone tissue for implant installation.

**Figure 2:**
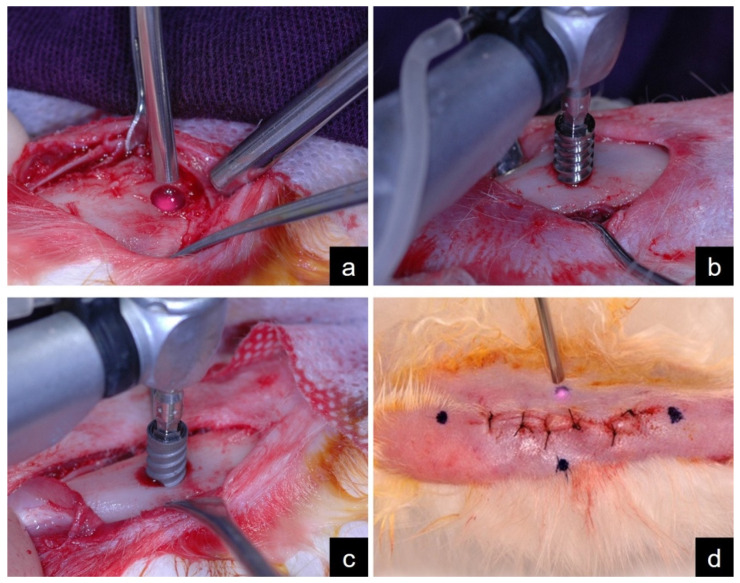
Experimental surgery: (**a**) PBM therapy in the surgical site (LMS and LTS groups); (**b**) Installation of machined surface implant; (**c**) Installation of surface-treated implant; (**d**) PBM therapy in four points after suture (LMS and LTS groups).

To prepare the surgical site, an electric motor (Driller BLM 350, São Paulo, Brazil) with a final speed of 1200 rpm and a 20:1 contra-angle (Kavo do Brazil, Joinville, Brazil) was used. Initially, an osteotomy with a lance drill was performed to break the cortical bone. Thereafter, helical drills were used, scaled as follows: 2.0/2.5 mm, 2.5/2.8 mm, 2.8/3.2 mm, 3.2/3.5 mm and 3.5/3.8 mm. All osteotomies were performed under constant irrigation with 0.9% saline solution (Darrow, Rio de Janeiro, Brazil) to avoid harmful heating in the region. After preparing the surgical site, the LMS and LTS groups received PBM therapy, and a low-level laser (GaAlAs laser) was applied (DMC Equipments, Whitening Lase II, São Carlos, Brazil) perpendicularly to the surgical site (Figure 2a) with a wavelength of 880 nm, a power of 100 mW, dose of 8.7 J/cm² and a duration of 25 s for an area of 1 cm² (pre-programmed dosimetry for implantology).

The machined (Figure 2b) and treated (Figure 2c) were installed at a speed of 20 rpm and with a fixed torque of 20 Ncm. The muscle suture was performed using absorbable polyglactin 910 thread (Vycril 4.0, Ethicon, Johnson Prod., São José dos Campos, Brazil) and a cutaneous suture using non-absorbable nylon thread (Ethicon 4.0, Johnson, São José dos Campos, Brazil). Postoperatively, the animals received intramuscular administration of Pentabiotic (0.1 mL/kg, Fort Dodge Saúde Animal Ltda, São Paulo, Brazil) and sodium dipyrone (1 mg/kg/day mg/kg, Ariston Indústrias Químicas e Farmacêuticas Ltda, São Paulo, Brazil) in a single dose. Subsequently, the LMS and LTS groups received PBM therapy perpendicularly at four different points (Figure 2d).

### 2.3. Resonance Frequency Analysis

Immediately after implant installation, the stability coefficient (ISQ) was measured using a resonance frequency analyzer (Ostell^®^ Instrument, Integration Diagnostics AB, Gotemburgo, Sweden). Measurements were performed in four different implant positions using a compatible transducer (SmartPegsTM Instrument, Integration Diagnostics AB, Gotemburgo, Sweden) that was screwed into the implant. Thus, four readings for each implant were obtained, and the arithmetic mean was calculated. After 21 and 42 days, the implants were reopened for a new frequency measurement by resonance using the Osstell^®^ device [22].

### 2.4. Biomechanical Analysis

Implants removed using a removal torque were connected to a digital torque (Data Tork CEM 3, Tohnichi Mfg. Co., Ltd., Tokyo, Japan). The necessary force in Ncm to break the interface formed between the bone and the implant was measured [22].

### 2.5. Analysis of Implant Surfaces

Prior to the experimental surgery and biomechanical analysis, the surface topography of the implants was analyzed using a scanning electron microscope (SEM model XL 30 TMP, FEG, Philips XL Series, with Oxford incaX-sight detector, Netherlands, 97), coupled to an EDX for the semi-quantitative analysis of the chemical composition of the surfaces prior to implant placement. After the removal of the implants using the removal torque, an evaluation of the newly formed bone tissue was performed [22] at 21 and 42 days postoperatively. A semi-quantitative analysis was performed for calcium (Ca), phosphorus (P) and oxygen (O).

### 2.6. Statistical Analysis

The analyses were performed using the software SigmaPlot 12.0 (Exakt Graph and Data Analysis). With respect to the sample size calculation, in order to estimate an appropriate number of animals, the removal torque value was used as the primary outcome. For this variable, a difference of 5% with a standard deviation of 2% was deemed as significant. Considering a power of 80% and setting alpha at 0.05, five implants per group were necessary in order to compare five different groups.

Group measurements are expressed as the mean ± standard deviation. The data obtained in each type of comparison were tested using the Shapiro–Wilk normality test, which confirmed the normal distribution of all data and analyses. Subsequently, data were subjected to statistical analysis of variance followed by a post hoc Tukey’s test for multiple comparisons between the groups (*p* < 0.05).

## 3. Results

### 3.1. Resonance Frequency Analysis

**Figure 3:**
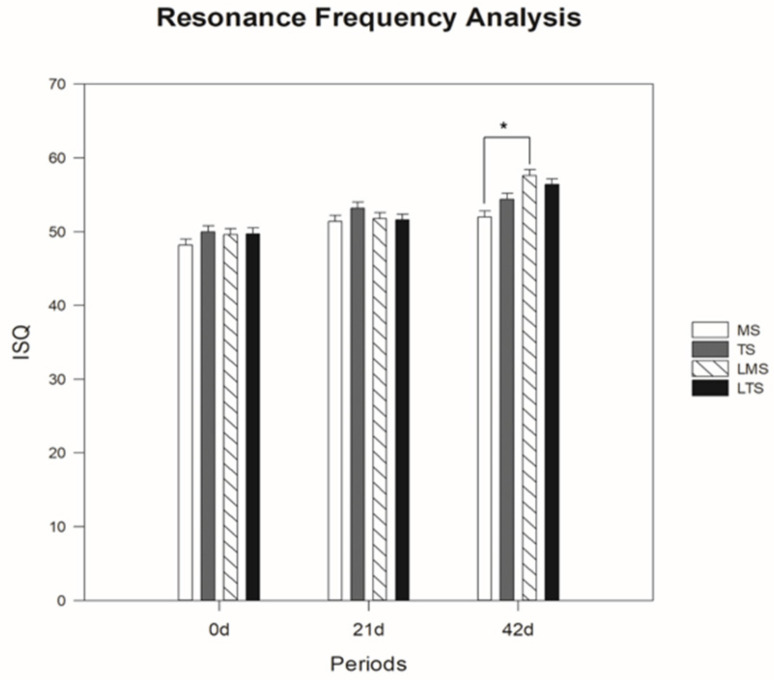
Mean values and standard deviation of the resonance frequency analysis between the groups in the periods of 0, 21 and 42 days (* *p* < 0.05).

The ISQ mean and standard deviation values for the MS group were 48.2 ± 2.59, 51.4 ± 2.41 and 52 ± 2.24 in the time intervals of 0, 21 and 42 days, respectively. For the TS group, the mean values were 50 ± 1.87, 53.2 ± 2.28 and 54.4 ± 0.89, respectively, in the same periods. The mean ISQ and standard deviation values for the LMS group were 49.6 ± 1.82, 51.8 ± 1.48 and 57.6 ± 0.89 in the time intervals of 0, 21 and 42 days, respectively. For the LTS group, the mean values were 49.7 ± 1.57, 51.6 ± 0.89 and 56.4 ± 1.34, respectively, in the same periods. When the effects of PMB therapy on the machined surface implants were evaluated, the mean resonance frequency values of the LMS implants were significantly higher (*p* < 0.001) than those of the MS implants at only 42 days (Figure 3).

### 3.2. Biomechanical Analysis

**Figure 4:**
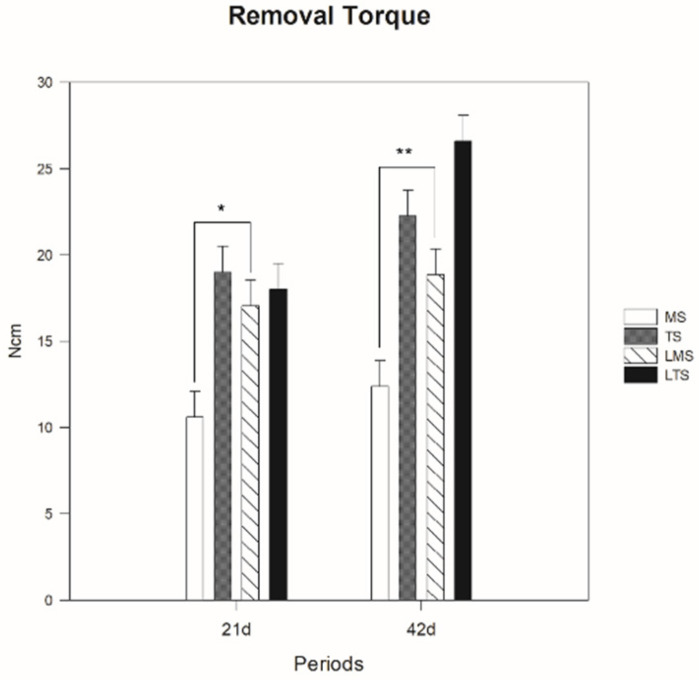
Mean values and standard deviation of removal torque between groups in the periods of 21 and 42 days. (*, ** *p* < 0.05).

The mean torque removal values in the MS group were 10.6 ± 1.23 and 12.38 ± 2.14 Ncm in the periods of 21 and 42 days, respectively, whereas those for the TS group were 19 ± 5.52 and 22.26 ± 1.61 Ncm, respectively, in the same periods. The mean torque removal values for the LMS group were 17.06 ± 4.64 and 18.84 ± 2.72 in the periods of 21 and 42 days, respectively, whereas those for the LTS group were 18 ± 2.56 and 26.6 ± 3.97, respectively, in the same periods. The modification of the surface increased the mean values of torque removal since TS was statistically higher than MS in the periods of 21 (*p* = 0.002) and 42 days (*p* < 0.001), and LTS was statistically higher than MS (*p* = 0.008) on day 21 and MS (*p* < 0.001) and LMS on day 42 (*p* = 0.005) (Figure 4). When comparing the effect of PBM therapy on the machined surface, the mean of the torque removal values of the LMS group was statistically higher than that of the MS group in the periods of 21 (*p* = 0.023) and 42 days (*p* = 0.023). However, the same effect of PBM therapy was not observed on the treated surface, since there was no statistical difference between TS and LTS at 21 (*p* = 0.965) or 42 days (*p* = 0.194).

### 3.3. Analysis of Implant Surfaces Prior to Placement

**Figure 5:**
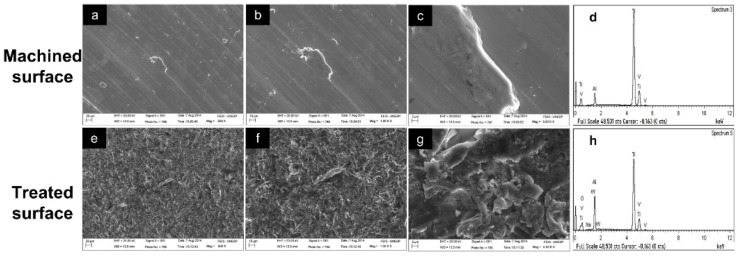
Topographic surface analysis of machined and treated surface implants prior to experimental surgery: (**a**–**c**) SEM: machined surface, 500×, 1000× and 5000×; (**d**) EDX: machined surface; (**e**–**g**) SEM: treated surface, 500×, 1000× and 5000×; (**h**) EDX: treated surface.

The machined surface showed a smooth surface topography with machining remainders (Figure 5a–c), whereas the treated surface presented a topography comprising a morphological pattern of subtraction with valleys of different sizes and depths (Figure 5e–g). EDX analysis showed peaks of Ti (titanium), Al (aluminum) and V (vanadium) in the machined surface (Figure 5d), whereas the treated surface showed peaks of Ti, Al, V and O (Figure 5h). The presence of oxygen in this group occurred as a result of implant surface treatment. 

### 3.4. Analysis of the Surfaces of the Implants Removed by Removal Torque

**Figure 6:**
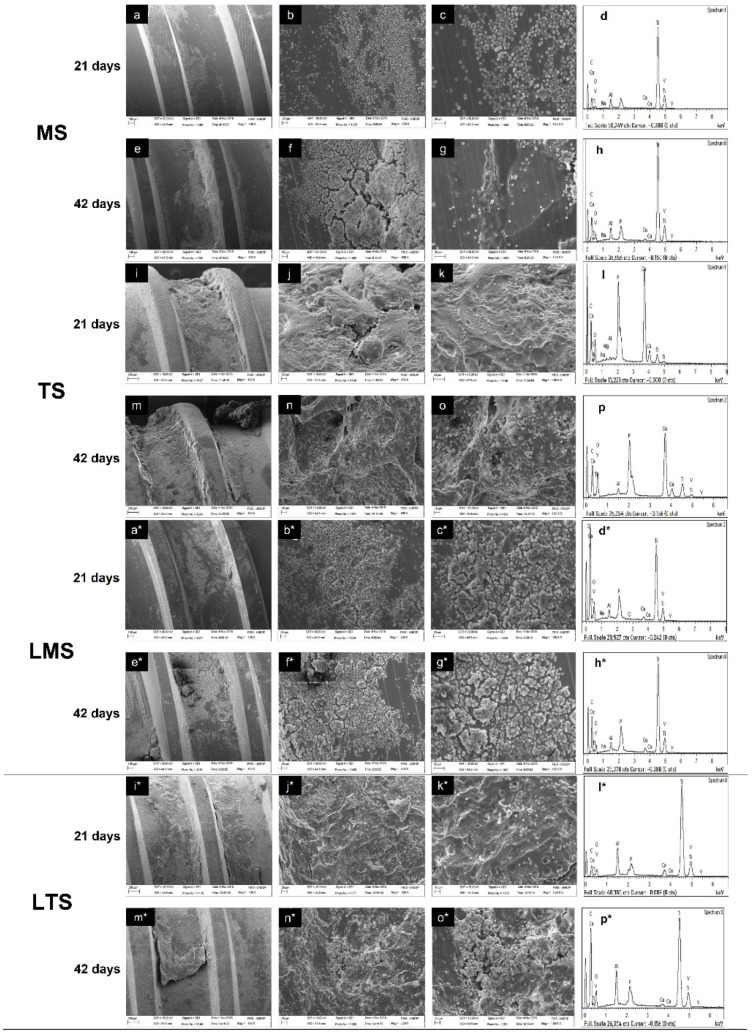
Topography surface analysis after torque removal. SEM (100×, 500× and 1000×) and EDX. (**a**–**d**) MS group at 21 days; (**e**–**h**) MS group at 42 days; (**i**–**l**) TS group at 21 days; (**m**–**p**) TS group at 42 days; (a*–d*) LMS group at 21 days; (e*–h*) LMS group at 42 days; (i*–l*) LTS group at 21 days; (m*–p*) LTS group at 42 days.

Regardless of the study period, the LMS group (Figure 6a*–c*,e*–g*) showed a higher level of bone coverage than the MS group (Figure 6a–c,e–g). In general, the surface of the implants removed by reverse torque at 42 days postoperatively in the LMS (Figure 6e*–g*), TS (Figure 6m–o) and LTS groups (Figure 6m*–o*) presented a higher level of bone coverage when compared to the MS group (Figure 6e–g). The EDX of all analyzed surfaces showed peaks in Ti, Al, V, O, Ca and P, revealing an increase over the study period when compared to the LMS, TS and LTS groups (Figure 6d*,h*,l,p,l*,p*) with the MS group (Figure 6d,h).

## 4. Discussion

In the present study, the values obtained by the resonance frequency analysis showed a statistical difference in the LMS group compared with the MS group at 42 days (*p* < 0.001). Regarding the removal torque analysis of the implants, the values obtained in the LMS group were significantly different from those in the MS group at 21 days (*p* = 0.023) and 42 days (*p* = 0.023). These results were found in previous studies published in vivo, which evaluated the removal torque of implants with or without PBM therapy, showing that laser therapy was advantageous, improving the resistance of the bone–implant interface and contributing to the osseointegration process [14,15,16,17,18,19,20].

PBM therapy has several therapeutic effects, including tissue biostimulation, allowing the acceleration of healing processes, bone repair and the attenuation of painful processes, among other beneficial effects [6,7,8,9]. The first study using a low-level laser for tissue biostimulation was described in 1971 [9]. From this study, several authors [5,10,13,14,23,24,25] have described the beneficial effects of PBM therapy, thus obtaining an effective and efficient treatment. These effects of PBM therapy in the present study were better observed in machined surface implants, since in the resonance frequency analysis in the comparison between treated surface implants at 0 days (*p* = 0.993), 21 days (*p* = 0.496) and 42 days (*p* = 0.301), as well as in the biomechanical analysis at 21 days (*p* = 0.965) and 42 days (*p* = 0.194), no statistical differences were observed between the TS and LTS groups. This finding reveals that the effects of PBM therapy were not sufficiently evident on the treated surface, since topographic modifications of the implant surface are considered an advance in the field of implantology, as they provide superior values of resonance frequency, removal torque and bone–implant interface [23,26,27]. Thus, the modification of the implant surface modulated the osseointegration process, and the PMB therapy did not increase the biological efficiency in this peri-implant repair process of the treated surfaces.

Previously published studies have demonstrated the efficacy of PBM therapy in animals compromised systematically, and it may have a more evident effect on unfavorable repair conditions [28,29,30]. This fact can be observed in the present study, as the effects of PBM therapy mainly on machined surface implants were identified, which is a surface that depends on the time for healing and requires greater care during the osseointegration process. SEM–EDX complemented the results found in the resonance frequency and biomechanics analysis, in which it was possible to observe that the treated surfaces of the implants presented a higher level of bone coverage and higher values of Ca, P and O than implants on the MS, mainly at 42 days.

Within the limitations of the present study, PBM therapy can be considered an adjunct treatment to obtain superior peri-implant bone repair, especially in patients with systemic or local diseases that can interfere with osseointegration, such as smoking or lower-quality bone tissue. Thus, the present study confirms the hypothesis that photobiomodulation (PBM) therapy with a wavelength of 880 nm has a biostimulating effect on peri-implant bone tissue and was evidenced mainly on the machined surface. However, additional analyses should be performed to study demineralized as well as mineralized tissues in-depth, including microCT, confocal laser and histomorphometric analysis, in order to validate and improve the results achieved by PBM therapy in peri-implant bone healing.

## 5. Conclusions

In view of the results obtained, PBM therapy modulated the osseointegration process, providing the biostimulation of the bone tissue peri-implant. The effect of PBM therapy was evidenced mainly on the machined surface.

## Data Availability

Not applicable.
